# An Unusual Case of Burning Mouth Syndrome in an Adult Male: A Case Report

**DOI:** 10.7759/cureus.44847

**Published:** 2023-09-07

**Authors:** Tharajan Gunendran, Payal Bai, Rashmi Subhedar, Uvesh Mansuri, Sakshi Jain

**Affiliations:** 1 Family Medicine, All Saints University, Roseau, DMA; 2 Public Health, Icahn School of Medicine at Mount Sinai, New York, USA; 3 Psychiatry, Seth Gordhandas Sunderdas Medical College (GSMC) and the King Edward Memorial (KEM) Hospital, Mumbai, IND; 4 Medicine, MedStar Union Memorial Hospital, Baltimore, USA; 5 Geriatrics, Hackensack University Medical Center, New Jersey, USA

**Keywords:** trigeminal nerve, p2x3, purinergic receptors, trpv-1, transient receptor potential vanilloid-1, oral pain, orofacial pain, glossodynia, bms, burning mouth syndrome

## Abstract

Burning mouth syndrome (BMS) is a rare disorder primarily affecting the oral mucosa and characterized by a chronic burning sensation without specific oral mucosal lesions. This paper presents a case of a 54-year-old adult male patient who complained of chronic burning mouth pain. The clinical diagnosis was made after excluding various differentials, including oral candidiasis, hairy oral leukoplakia, gastroesophageal reflux disease, oral lichen planus, local infective processes, and nutritional deficiencies. Physical examination did not reveal specific signs or lesions related to BMS; however, considering the patient's signs, symptoms, and the exclusion of other possibilities, a possible diagnosis of BMS was considered. The patient was evaluated in an outpatient setting, and management was conducted in this setting to reduce patient costs. This presentation is considered rare, as the disorder predominantly affects postmenopausal females, and most proposed theories behind its pathophysiology revolve around estrogen-mediated modulation of pain receptors. Currently, diagnostic and management criteria for BMS may vary and continue to evolve. The management of this patient focuses on patient education and routine follow-up. This case report presents the management of this particular case, along with a review of other proposed management options.

## Introduction

Burning mouth syndrome (BMS) is a chronic condition that arises spontaneously, characterized by a continuous burning sensation in the oral mucosa, with or without lip extension, in the absence of a specific oral lesion. It is associated with abnormalities in gustation, xerostomia, and oral mucosal pain. The International Association for the Study of Pain (IASP) defines BMS as "an independent nosological entity characterized by unremitting oral burning or similar pain in the absence of detectable oral mucosal changes" [1-2]. Estimating the prevalence rates of BMS in the general population can be challenging due to significant variations in the literature. However, it is evident that there is a predisposition to peri/postmenopausal females [3]. A case-control study based on patient records from the Oral Pathology Service at the UW School of Dentistry over a seven-year period indicated a BMS prevalence rate of 0.99% (32 BMS patients/3,242 patient records), with females being more commonly affected and the majority of patients in their sixties [4]. A population study using medical records from the Rochester Epidemiological Project, recording newly identified BMS diagnoses from January 1, 2000, to December 31, 2010, revealed 169 incident cases, with an annual age- and sex-adjusted BMS incidence rate of 11.4 per 100,000 person-years. The age-adjusted incidence was significantly higher in women than in men (18.8 (95% CI, 15.6-22.9) per 100,000 person-years versus 3.7 (95% CI, 2.6-5.7) per 100,000 person-years; p<0.001). The results indicated that postmenopausal women aged 50 to 89 years had the highest incidence of the disease, with the peak being observed in women aged 70 to 79 years (70.3 per 100,000 person-years) [5]. A meta-analysis conducted in 2021, including 18 studies (9 population studies and 9 clinical studies), revealed a BMS prevalence varying from 1.73% in the general population to 7.72% in the clinical population, with women over 50 years being more commonly affected at a 3:1 ratio compared to men [6]. A wide range of theories has been proposed on symptom occurrences and etiology; however, in a clinical setting, the diagnosis and approach to disease management are still unclear [7-8].

## Case presentation

A 54-year-old male patient with a past medical history of treated tuberculosis infection 20 years ago presented to the medical office with a complaint of pain inside his mouth. The patient mentioned that the pain started one year ago and was felt throughout his entire mouth without lip involvement. He described the pain as a burning sensation similar to eating chili peppers and associated it with mouth dryness. Drinking water helped alleviate the pain, and he denied any aggravating factors or radiation of pain to other parts of his body. The patient reported that the pain was worse in the morning but gradually improved as the day progressed. On a pain rating scale from 1 to 10, the patient scored the pain at 5/10 at its worst. He also mentioned that six months ago, he presented with similar symptoms at another outpatient clinic in Pakistan and was prescribed oral Fluconazole 100 mg once a day and Nystatin mouthwash for two weeks, which he completed. However, the prescribed regimen did not relieve his symptoms. The patient denied a history of HIV infection, sexually transmitted diseases, heartburn, diabetes mellitus, history of allergies, recent dental procedures, nocturnal cough, difficulty swallowing, or pain with swallowing. He also confirmed not taking any medications. During the examination, the patient appeared mildly anxious, and his ambulatory blood pressure was 129/82 mmHg, heart rate 106 beats per minute, and temperature 98.6 degrees Fahrenheit.

**Figure 1:**
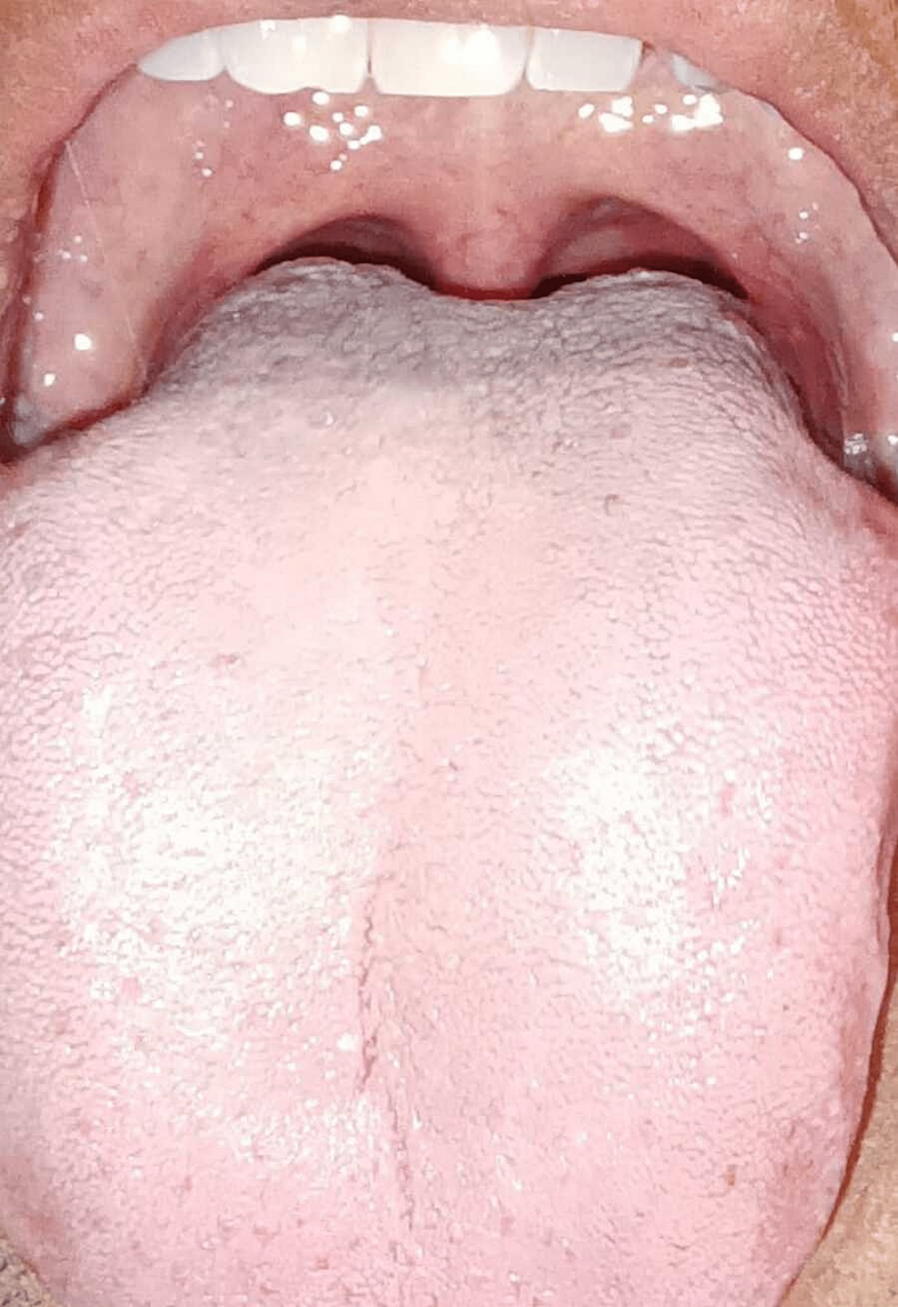
Clinical presentation of burning mouth syndrome in a patient

As per Figure 1, enlarged papillae were noted on the posterior aspect of the tongue while the tonsils appeared normal. A yellowish, thin coat was observed over the tongue diffusely, but no hairy projections or thick coating were seen. Initial laboratory testing, including human immunodeficiency virus (HIV), hemoglobin A1c (HBA1c), complete blood count (CBC), complex metabolic profile (CMP), fasting lipid panel, antinuclear antibodies (ANA), anti-Sjogren syndrome A (SSA/Ro), anti-Sjogren syndrome B (SSB/La) antibodies, rheumatoid factor (RF), iron panel, zinc, vitamin B12, folate, riboflavin, and thiamine levels, yielded unremarkable results. The patient completed the Patient Health Questionnaire-2 (PHQ-2), Patient Health Questionnaire (PHQ-9) (PHQ-9), and General Anxiety Disorder-7 (GAD-7) assessments in-office, scoring 2 points for depression and 8 points for mild anxiety. During patient counseling, the multifactorial nature of the diagnosis and the lack of a clear pathophysiological process and specific medical regimen for treatment were discussed. The potential causative relationship between chronic anxiety and burning mouth syndrome was also addressed. Through shared decision-making, a referral for cognitive-behavioral therapy with psychologists within the patient's area code was offered, and the potential benefits of regular follow-ups in the office and supportive therapy were discussed. The patient was advised to follow up in one month.

## Discussion

Burning mouth syndrome (BMS) is a chronic condition characterized by a burning sensation of the oral mucosa for which no cause can be found. The anterior part of the tongue is most commonly affected, followed by the labial mucosa and occasionally the palate. The burning pain is often accompanied by tingling or numbness and the sensation of dryness in the mouth. Reduced taste intensity and a bitter or metallic taste are experienced in about two-thirds of those with BMS. Despite these, the oral mucosa salivary flow rates are normal [9-12]. BMS is classified clinically into three subtypes: Type 1 BMS is associated with underlying systemic diseases such as nutritional deficiencies and diabetes mellitus, Type 2 BMS is associated with underlying psychological disorders, and Type 3 BMS is related to allergic reactions or local factors [6]. Common features of BMS include but are not limited to a burning, painful sensation in the mouth, often associated with dysgeusia and xerostomia despite normal salivation. Classically, symptoms are better in the morning, worsen during the day, and typically subside at night [4]. Though the pathogenesis of BMS isn’t known, theories have been proposed that psychogenic factors can play a role. It is thought that some pain-modulating neural pathways descending from the cortex, hypothalamus, midbrain, and medulla are influenced by emotional states, such as excitement, stress, anxiety, and depression, and these can either potentiate or suppress spinal nociceptive pathways. It’s also thought that these factors can spontaneously induce nociceptive signals without peripheral input as in the case of BMS [11-13]. The presentation of BMS can overlap with Sjogren syndrome (SS); however, distinguishing factors include the presence of ocular and salivary histopathological findings in SS not seen in BMS, and serological findings with anti-Ro, anti-La, ANA, and RF are negative in BMS. In SS, it is considered salivary dysfunction while in BMS, it is perceived as xerostomia with normal salivary function [14-15]. It has further been proposed that the downregulation of central dopaminergic pain-inhibitory pathways can be seen in persons experiencing anxiety or depression, which are both associated with dysregulation of central mood-mediating dopaminergic pathways [11-13]. Theories have also been proposed that both peripheral and central neuropathies have a hand in the pathogenesis of BMS. Multiple tongue mucosal biopsy studies in patients with BMS have shown decreased intraepithelial nerve fiber densities, immunohistochemical staining has shown a significant increase in transient receptor potential vanilloid-1 receptors (TRPV1), nerve growth factor (NGF) receptors, and purinergic receptors (P2X3). TRPV1, in particular, is found physiologically on the trigeminal nerve peripheral nociceptive terminals, which is significant in that the oral mucosa receives its innervation from the maxillary and mandibular branches of the trigeminal nerve [9-14]. TRPV1 is also seen centrally in the dorsal root ganglion and trigeminal ganglia. It is thought that TRPV1 is responsible for the transmission of heat sensation from the oral mucosa in response to heat or hot taste (capsaicin) [14-16]. Recent studies have shown that estrogen plays a significant role in understanding pain perception and gender disparities by influencing TRPV1 and P2X3 receptors, which are key components. Although some studies have indicated that estrogen increases pain through TRPV1 activation and decreases P2X3 activity, recent data suggest that it may also have the potential for pain reduction by reducing nerve growth factor (NGF) levels that affect TRPV1 receptor functions. Notably, estrogen has been observed to decrease NGF expression in rat chondrocytes, which supports previous research and suggests a complex and nuanced role in pain modulation that needs further investigation.

**Figure 2:**
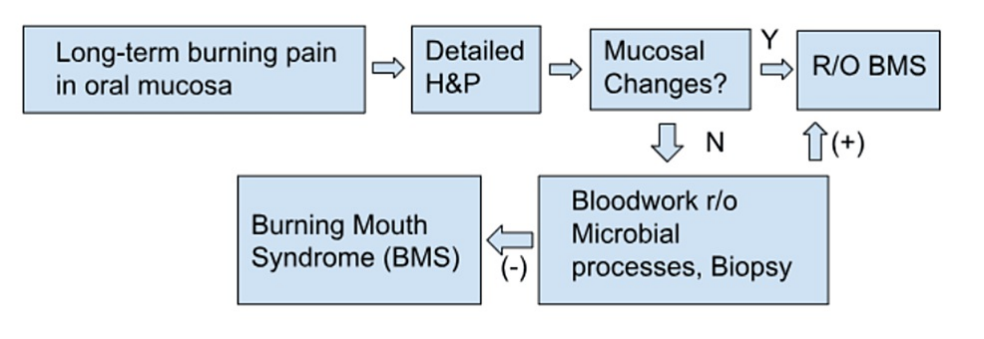
Clinical diagnostic algorithm for BMS diagnosis BMS - burning mouth syndrome, r/o - rule out

As per Figure 2, the diagnosis of BMS was made in an outpatient office, clinically based on the exclusion of differentials. The patient’s lack of immunosuppression history or recent antibiotic use, along with the failed empiric treatment of candida, helps rule out oral candidiasis. While the lack of hairy projections bilaterally on the lateral borders of the tongue rules out oral hairy leukoplakia, the lack of heartburn symptoms or nocturnal cough rules out gastroesophageal reflux disease. Nutritional deficiencies and autoimmune disorders were ruled out with unremarkable laboratory testing. Lack of associated buccal, gingival, and/or skin lesions helps rule out oral lichen planus. No fever/chills, local lesions, discharge, or leukocytosis on laboratory testing rules out local infective processes. A significant GAD-7 score, despite unremarkable PHQ-2 and PHQ-9 scores, suggests coexisting anxiety with a possible diagnosis of Type 2 BMS. Further investigation to contribute to the diagnosis was not performed but, if desired, includes objective measurements of salivary flow rates and taste function, collections of oral secretions for culture to rule out bacterial, viral, or fungal processes, patch testing to rule out underlying allergic processes, and gastric reflux studies [3]. It is recommended that patients should be told that BMS is a complex disorder without a cure and that treatment is purely symptomatic to give patients realistic expectations moving forward [17-18].

As per Table 1, potential treatment options include capsaicin, which is a component of chili peppers. It interacts with TRPV1 ion channels to induce constant depolarization and transmission, which is thought to cause eventual reversible axonal degeneration of the peripheral nociceptive fibers and consequent reduction in burning sensation. Clonazepam, a gamma-aminobutyric acid (GABA) agonist, can activate pain-inhibitory pathways in the spinal cord and peripheral nociceptors to provide analgesia. It also has anxiolytic properties that can decrease the underlying anxiety/stress if present. Antidepressants are given to patients with diagnosed anxiety/depressive disorders and comorbid BMS. Alpha lipoic acid, a naturally occurring antioxidant in the body, has been shown to be effective in treating diabetic neuropathy and has been proposed to yield positive results in patients with BMS. Anticonvulsants like gabapentin can be given as well since it is commonly used in patients with neuropathic pain [11-12]. A systematic review and network meta-analysis (NMA) of previously conducted randomized control trials (RCTs) comparing the effectiveness of treatments for pain relief in patients with BMS was done using RCTs from the Cochrane Database of Systematic Reviews and Central Register of Controlled Trials (CENTRAL), Medline, Embase, Scopus, Web of Science, ClinicalTrials.gov, the International Clinical Trials Registry Platform, and ProQuest Dissertation and Theses databases. In total, 44 RCTs involving 2283 patients were taken. The authors concluded, “Among all tested treatments, only Clonazepam is likely to reduce the pain of BMS compared with placebo. The majority of other treatments had low and very low certainty, mainly due to imprecision, indirectness, and intra-sensitivity” [10]. Cognitive-behavioral therapy has been an effective form of treatment for BMS in previous controlled trials [14-15]; however, it is thought that 12-16 sessions are necessary to complete a CBT course, which is difficult due to its high cost of treatment [17]. Drug treatments, including antidepressants, dietary supplements, hormone-replacement therapy, and psychological support, have been proposed; however, the management of BMS remains unsatisfactory for patients. The prolonged pain and trials of unsuccessful treatments associated with BMS can affect a patient's mood and induce or reinforce psychiatric disorders such as anxiety, depression, and cancerophobia, possibly decreasing their quality of life [7].

**Table 1:** Pharmacological treatments for burning mouth syndrome GABA - gamma-aminobutyric acid, TRPV-1 - transient receptor potential vanilloid-1

Medications	Description
Capsaicin	Component of chili peppers, interacts with TRPV-1 ion channels, reversible axonal degeneration peripheral nociceptive fibers in oral mucosa
Clonazepam	GABAergic agonist, activates pain inhibitory pathways in the spinal cord and peripheral nociceptors, anxiolytic
Alpha Lipoic Acid	Naturally occurring antioxidant, free radical breakdown
Gabapentin	GABA analog, inhibitory effect on nociceptive fibers

## Conclusions

In the described case, this 54-year-old male patient presented in an outpatient setting with burning mouth pain associated with dry mouth for the past year and was clinically diagnosed based on the exclusion of differentials. Management involved patient education and proposed cognitive behavioral therapy. Pharmacological treatments were discussed but not proposed to the patient during the visit. At the one-month follow-up appointment, the patient reported improvement indicating the possibility that patient education on the disorder and reassurance slightly improved the patient’s ability to tolerate his symptoms. Observation and supportive care in management helped decrease patient costs and improve the quality of life of this patient with burning mouth syndrome. This, alongside a clinical diagnosis, helped further decrease patient costs in place of available more expensive laboratory testing. The limitations of the outpatient setting compared to inpatient directed the diagnostic approach, however, it is possible that this direction itself leads to an overall positive impact on a patient with a disorder where management strategies remain uncertain. With the passage of time and with additional cases reported, more distinct diagnostic and management strategies may be established.
